# ﻿Resolving the taxonomy of *Leea trifoliata* (Leeaceae, Vitales): Reinstatement and typification

**DOI:** 10.3897/phytokeys.266.173605

**Published:** 2025-11-21

**Authors:** Amrutha Athalappil, Ayilliath Kuttiyeri Pradeep, Jun Wen, Nikhil Krishna

**Affiliations:** 1 Department of Botany, University of Calicut, Kerala 673 635, India University of Calicut Kerala India; 2 Department of Botany, National Museum of Natural History, Smithsonian Institution, Washington, DC, 20013-7012, USA National Museum of Natural History, Smithsonian Institution Washington United States of America; 3 Regional Ayurveda Research Centre, Dimapur, Nagaland 797112, India Regional Ayurveda Research Centre Dimapur India

**Keywords:** IUCN, *
Leea
*, neotype, Old World tropics, Vitaceae

## Abstract

*Leea trifoliata* M.A.Lawson has long been considered conspecific with *L. compactiflora* Kurz. Detailed examination of live materials and herbarium specimens suggests that both species are distinct, differing in habit, leaf architecture, and inflorescence and bract morphology. This contribution presents morphological evidence for the reinstatement of *L. trifoliata*, including its taxonomic description, illustration, distribution maps, notes on habitat, and conservation status assessment using the IUCN criteria. In addition, A neotype was selected for *L. compactiflora*.

## ﻿Introduction

The economically important grape order Vitales has two families, Leeaceae and Vitaceae (Wen 2007a, 2007b), which have sometimes been recognized as subfamilies Leeoideae Burmeister and Vitoideae Eaton of Vitaceae (APG III 2009; APG IV 2016). The order is widely distributed in the tropical and temperate zones, with Leeaceae being monotypic, consisting of the genus *Leea* D.Royen ex L. Many taxonomists (e.g. Planchon 1887; Gagnepain 1950; Suessenguth 1953; Ridsdale 1974, 1976; Naithani 2000; Latiff 2001; Ren et al. 2003; Chen and Wen 2007; Wen 2007a) have long treated *Leea* in its own family Leeaceae, due to its usually shrubby to tree habit, absence of tendrils, presence of petiolar stipules, development of a complex floral disc attached to a polypetalous corolla, the absence of nectar production, the presence of secondary septa in the ovary, the locules of the ovary each having only one ovule, as well as the distinct wood and palynological characters. The similarity of *Leea* and members of Vitaceae has been noted in terms of seed and embryo characters as well as the occurrence of pearl glands on the vegetative organs (Ridsdale 1974; Wen 2007a, 2007b).

*Leea* consists of c. 45 species (POWO 2025) and is distributed throughout the Old World tropics, ranging from Africa and Madagascar to Fiji (Ridsdale 1976). The Malesian Archipelago has the highest number of species and endemism, with 25 species in this region (Ridsdale 1974, 1976; Lok et al. 2011). Within the genus, the circumscription of many of the species remains controversial (Molina et al. 2013), and the only significant taxonomic work on the genus is the revision of Leeaceae by Ridsdale (1974, 1976). However, Ridsdale’s studies were mainly based on herbarium specimens available at different herbaria. He himself recommended a complete field-based study to resolve several species complexes in the genus.

During the botanical expedition to northeastern India, we were able to collect a highly distinct trifoliate plant from Arunachal Pradesh, India. After conducting an extensive literature survey pertaining to this genus (e.g. Lawson 1875; Clarke 1881), we came across *Leea trifoliata*, which was first described by Lawson in 1875 in Hooker’s Flora of British India. Lawson (1875) described this species as a small, wiry plant, 1–2 feet tall and noted that it was allied to *Leea aspera* Wall. We were able to locate the syntype material of Jenkins & c. (K000736385 & K000323729), Booth (K000323730), and Griffith (K004252755, P02342863) in K and P. Additionally, our examination of the extensive herbarium specimens from India (AHMA, ARUN, ASSAM, BSHC, BSI, BSID, CAL, CALI, MH, PBL, RHT and TBGT) and the United States National Herbarium (US), and other collections virtually (A, BM, BR, G, GH, K, L, MICH, MO, NY, P, SING & U), enabled us to locate and compare several historical collections of *L. trifoliata*.

*Leea trifoliata* was synonymized under *L. compactiflora* by Ridsdale (1974). Ridsdale adopted a broader species concept for most of the species. *Leea trifoliata* is restricted only to India and Bhutan. However, *Leea compactiflora*, as described by Kurz (1873) from Martaban, Myanmar, is a shrubby species distributed in India, Bhutan, China, Laos, Myanmar, Nepal and Vietnam. For the type material of *L. compactiflora*, we searched all possible herbaria, especially the sheets deposited by Kurz. However, we were unable to locate a specimen by Kurz collected from the type locality, Martaban or even from Myanmar. In his revision, Ridsdale (1974) was also unable to locate the specimen of Kurz. Hence, we need to typify the name.

Therefore, the aim of this paper is to examine the collections and morphology of both *L. trifoliata* and *L. compactiflora* to reevaluate the taxonomic status of *L. trifoliata* and to neotypify the name *L. compactiflora*.

## ﻿Materials and methods

### ﻿Materials

The materials for the present study were gathered during the field trip to northeastern India. The taxonomic key provided by Ridsdale (1974, 1976) and other literature dealing with this genus (Lawson 1875; Clarke 1881; Gamble 1918; Naithani 2000) as well as a large number of specimens were studied from various herbaria such as AHMA, ARUN, ASSAM, BSHC, BSI, BSID, CAL, CALI, MH, PBL, TBGT, A, BM, BR, G, GH, K, KUN, L, MICH, MO, NY, P, SING, U and US (Acronyms as per Thiers 2024 continuously updated) to assess the taxonomic status of *L. trifoliata*.

### ﻿Morphological analyses, distribution map, conservation assessments and typification

The descriptions were prepared based on the live materials collected from the field and materials planted in Calicut University Botanical Garden, and by examining the herbarium specimens from ASSAM, ARUN and US herbaria. The specimens were examined, and the photomicrographs were taken using an M80 Stereomicroscope (Leica, Mannheim, Germany) attached with a Leica MC 190 HD camera with Leica Application Suite Version 4.12. To take the field photographs, Nikon D3400 and Nikon D750 cameras (Nikon, Japan) were used. The descriptions were prepared by using the terminology of Ridsdale (1974, 1976), Stearn (1983), Harris and Harris (1995), Simpson (2019), and Kew Plant Glossary (Beentje 2016). A micro-tip pen (Rotring variant) equipped with 0.1 and 0.2 points was used for the preparation of the illustration. The distribution map was prepared using the software QGIS version 3.24.3 (QGIS 2022). During the field visits, the threat was thoroughly investigated. Extent of Occurrence (EOO) and Area of Occupancy (AOO) were calculated using Geocat (http://geocat.kew.org/), and conservation assessment was performed using IUCN Standards and Petitions Committee (Version 15.1 2022). For the selection of types, the most suitable and informative specimens were chosen in concurrence with the protologue and adhering to the provisions of the Madrid Code (Turland et al. 2025).

### ﻿Results and discussion

**Figure 1.**
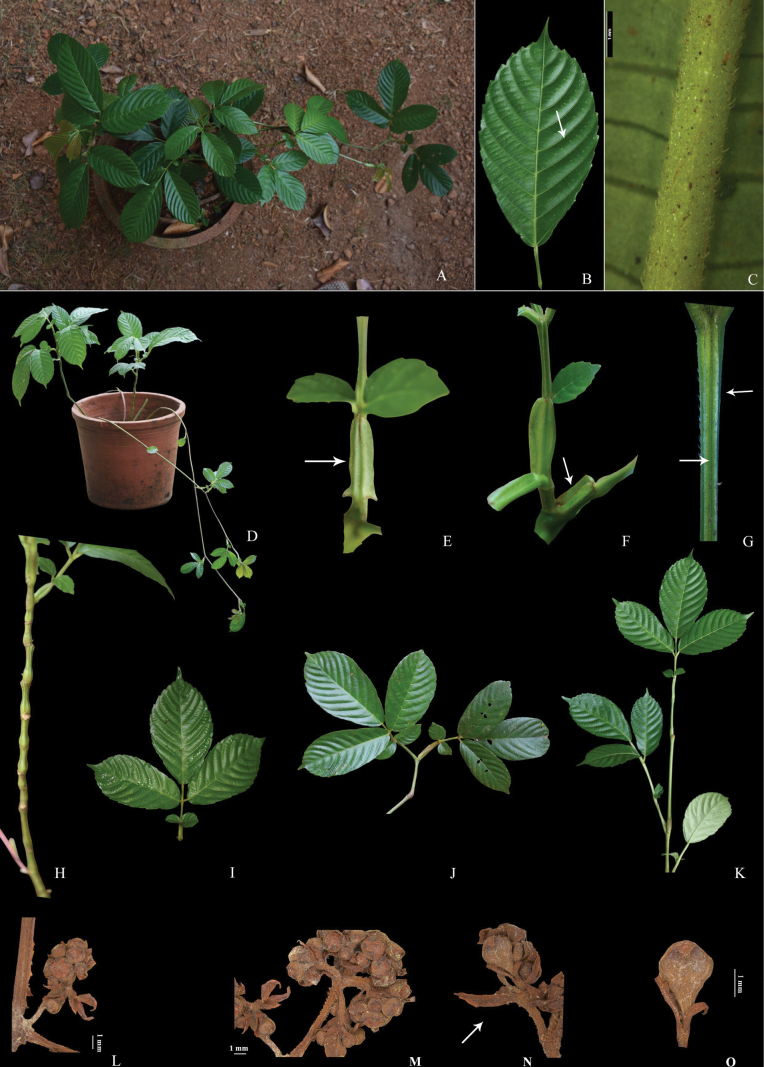
*Leea trifoliata* M.A.Lawson: **A, D.** Habit; **B.** Adaxial surface of leaflet showing trichomes; **C.** Abaxial surface of leaflet showing trichome on nerves; **E, F.** Petiolar stipules; **G.** Rachis showing wings; **H.** Stem; **I–K.** Leaves; **L–N.** Inflorescences with bracts; **Fl**ower Bud.

**Figure 2.**
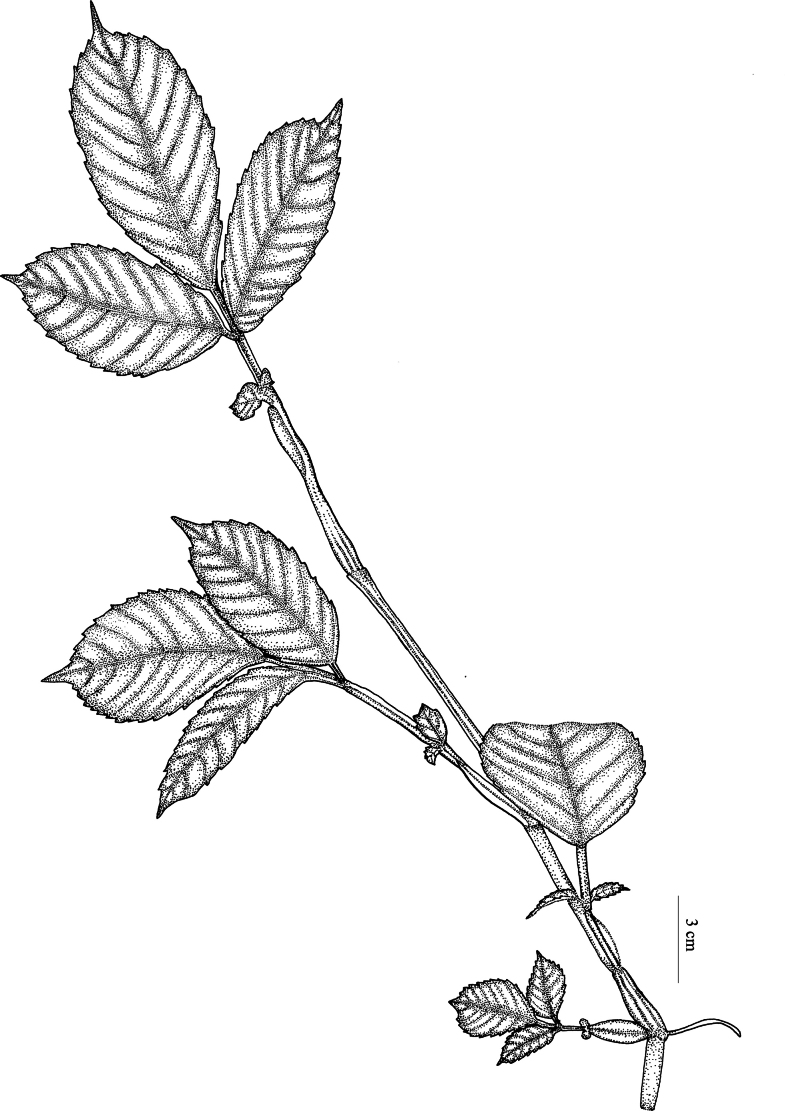
A twig of *Leea trifoliata* M.A.Lawson (Drawn by Amrutha A. from Amrutha 159851).

Clarke (1881) recognized *Leea trifoliata* and treated it in the series *Rubriflorae*, which is characterized by red-colored flowers, in his revision of Indian *Leea*, but admitted he was not sure about the color of the corolla. Subsequently, Kanjilal and Das (1936) included this species in the Flora of Assam. However, Ridsdale (1974) synonymized *L. trifoliata* under *L. compactiflora*. An extensive field exploration of both *L. trifoliata* and *L. compactiflora*, together with herbarium examinations, supports the distinctiveness of both species. *Leea trifoliata* differs from *L. compactiflora* by its habit, leaf architecture, petiolar stipule, characteristics of petiolule and rachis, size of inflorescence and shape and size of bracts. *Leea trifoliata* is a weak herbaceous species, and the remaining species of the genus are subshrubs, shrubs, to small trees. *Leea trifoliata* has a unique leaf architecture with mostly trifoliate or 1-pinnate leaves that bear a basal pair of highly reduced leaflets (Figs 1, 2), consistently distinct from those of other members of the *Leea*. We thus reinstate *L. trifoliata* and provide detailed comparisons of the two species in Table 1.

**Table 1.** Morphological comparisons of *L. trifoliata* and *L. compactiflora*.

Characters	*L. trifoliata*	*L. compactiflora*
Habit	0.8–1 (–2.5) m tall, weak, herbaceous, wiry plant	Over 1 m straggling or erect shrub
Leaf architecture	Mostly trifoliate or 1-pinnate, with a basal pair of leaflets always much reduced	Pinnate to bipinnate leaves
Indumentum on leaflets	One row of stiff hairs between nerves on the upper surface	Glabrous
Petiolule and rachis	Petiolules and rachises winged	Petiolules and rachises not winged
Petiolar stipule	Expanding the full length of the petiole, 2–2.5 cm long	Half of the length of the petiole, 4–8 cm long
Inflorescence size	Up to 4 cm long	8–22 cm long
Bracts	Lanceolate-linear	Broadly ovate
Calyx	Glabrous	Hispid

### ﻿Taxonomic treatment

#### ﻿ Leea trifoliata

Taxon classificationPlantae

M.A.Lawson, Hook.f. (Ed), Fl. Brit. India 1(3): 666. 1875.

C3642DC9-854E-576A-A573-7DE313107501

Figs 1, 2

Leea trifoliata M.A.Lawson, Hook.f. (Ed), Fl. Brit. India 1(3): 666. 1875; C.B.Clarke, Trimen’s J. Bot. 19: 163. 1881; Kanjilal & Das, Fl. Assam 1(2): 305. 1936. Type: INDIA. Assam, s.d., Jenkins & c. s.n. (lectotype, designated by Ridsdale 1974, pg. 88: K [K000736385]; isolectotype: K [K000323729]).

##### Description.

Perennial weak herb, 0.8–1 (–2.5) m high; roots non-tuberous. Stems erect or wiry, jointed, warted, glabrous; internodes furrowed, green; nodes swollen; pearl glands globose, white. Leaves alternate, trifoliate or 1-pinnate with two rudimentary pairs of leaves at base; petioles 7–21 mm long, slightly bulged, sheathing the stem, hairy at base, green. Petiolar stipules narrowly winged, expanding the full length of the petiole, 2–2.5 cm long; stipular scars triangular; rachises 1–4 cm long, channeled and slightly winged on upper surface, hispid on margin, green. Leaflets opposite, elliptic oblong or obovate, distal leaflets lamina: 5.5–13 × 2.7–7.5 cm, lateral leaflets lamina: 4.5–12.5 × 2–6 cm, one row of stiff hairs in between nerves on upper surface, mature leaves green on upper surface and pale green on lower, base cuneate, margins serrate, apex cuspidate caudate, abruptly acuminate; petiolules: central 1–2.5 cm long, lateral 0.2–0.7 cm long, channeled and winged on upper surface, green; secondary veins 7–16 pairs, 3-nerved at base, strigose on lower surface, vein raised on abaxial side. Inflorescences terminal or leaf-opposed, compound cyme, up to 4 cm long, axis hispid. Bracts 1.8–2.7 mm long, lanceolate-linear, pubescent. Calyx campanulate, c. 3.5 mm long, 5 lobed, each lobe c. 1–1.5 mm long, apex acute; corolla lobes 5, 2.7–2.8 × 1.1–1.3 mm, slightly hooded at apex, margin entire. Floral disc 1.1–1.5 mm long. Ovary 4–6 locular. Fruits noted as black in Lawson (1875).

##### Flowering and fruiting.

June–December.

##### Habitat.

This species mostly grows on roadsides and forest margins. They grow along with *Adiantum* sp. (Pteridaceae), *Impatiens minor* (DC.) Bennet, *Impatiens balsamina* L. (both Balsaminaceae) and some grasses.

##### Distribution.

**Figure 3.**
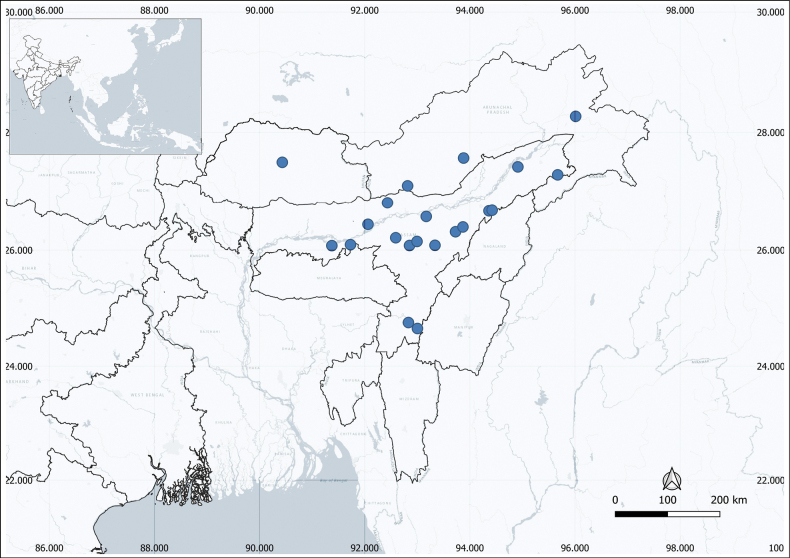
Distribution of *Leea trifoliata* M.A.Lawson in northeastern India and Bhutan (drawn using QGIS version 3.24.3).

Bhutan, India (Assam & Arunachal Pradesh). Fig. 3.

##### Specimens examined.

**Bhutan.** s.d., Booth s.n. (K000323730). **India.** • **Arunachal Pradesh**: Mishmi hills, s.d., Griffith 1344 (K004252755, P02342863); • East Kameng district, Pakke Wildlife Sanctuary, 25 Apr 2012, B.B.T. Tham 35028 (ARUN); • Kamle district, Raga, 27°47'59.4"N, 94°04'29.7"E, 16 Aug 2018, Amrutha 159851(CALI); • **Assam**: s.d., Masters s.n (K004252752); • Cachar district, Bhuban Hills, Oct 1978, 74142 (ASSAM); • Cachar, June 1874, R.L. Keenen s.n. (K004252761); • Darrang district, Aka Hills, s.d., N.L. Bohr 17378 (ASSAM); • Dibrugarh district, Dibru Reserve, eastern part, 20 Oct 1960, G. Panigrahi 21564 (ASSAM); • Golaghat district, s. loc, 300 ft, May 1891, Dr. King’s collectors s.n. (US01151426, K004252756, P02342865, L 0744660); • Jorhat district, Gibbon Sanctuary, Camp-2, 12 June 2010, Ranjit Daimary 122699 (ASSAM); • Ibid., Camp-4, 26 Feb 2011, Ranjit Daimary 121691 (ASSAM); • Kamarup district, Moyang Residence, 09 Apr 1915, U. Kanjilal 5449 (ASSAM); Kamarup Metropolitan district, Guwahati, April 1902, A.C. Chatterjee (P02342864, LY0106862, MPU703118); • Karbi Anglong district, • Garampani Wildlife Sanctuary, 24 Nov 1975, P.K. Hajra 65058 (ASSAM); Kaliani, 18 June 1963, D.B. Deb 35116 (ASSAM); • Nagaon district, Laopani, 28 Aug 1978, P. Chakraborty 78984 (ASSAM); • Sonitpur district, Batasipur, 13 May 1947, M.M. Srinivasan 22253 (ASSAM); • Kanchanjuri, Kazhiranga, 12 Aug 1973, P.K. Hajra 52836 (ASSAM); • Tinsukia district, Margherita Forest, 22 June 1938, G.K. Duka 17010 (ASSAM).

##### Conservation status.

*Leea trifoliata* is restricted to two northeastern Indian states, Assam and Arunachal Pradesh, and one location in Bhutan. This species prefers to grow in open areas adjoining evergreen forests. The Extent of Occurrence (EOO) is calculated to be 114,777 km2, and the Area of Occupancy (AOO) is 84 km2. Considering its large Extent of Occurrence, this species does not fall into any threat category. Despite this, the Area of Occupancy is small, and each population has fewer than five mature individuals. Due to its narrow range of distributions and small AOO, this species could be categorized at present as Endangered (EN), B2b(ii,iii) according to the IUCN Standards and Petitions Committee (2022).

##### Notes.

In the protologue, Lawson (1875) described the anthers as distinct, and the fruits were black.

Ridsdale (1974) typified the name *L. trifoliata* by selecting the Jenkins & c. material from Assam as the lectotype. We were able to trace two sheets of Jenkins & c. from the Kew herbarium (K000736385 & K000323729). However, Ridsdale has attached a slip to the sheet with barcode K000323729 stating that it is the lectotype. Hence, the other sheet (K000736385) becomes the isolectotype.

#### ﻿ Leea compactiflora

Taxon classificationPlantae

Kurz, J. Asiat. Soc. Bengal, Nat. Hist. 42(2): 65. 1873

307B0A93-379D-5E00-B002-F58EB30B6027

Figs 4, 5

Leea compactiflora Kurz, J. Asiat. Soc. Bengal, Nat. Hist. 42(2): 65. 1873, Forest Fl. Burma 1: 279. 1877; C.B.Clarke in Trimen’s J. Bot. 19: 163. 1881; Ridsdale, Blumea 22: 88. 1974; J.Joseph, Fl. Nongpoh and vicinity 72. 1982; Harid. & R.R.Rao, Forest Fl. Meghalaya 248. 1985; A.S.Chauhan, Contr. Fl. Namdapha 140. 1996; Hajra et al., Mat. Fl. Arunachal Pradesh 1: 318. 1996; B.D.Naithani in N.P.Singh et al. (Ed), Fl. India 2000; R.Shanpru in N.P.Singh et al. (Eds), Fl. Manipur 1: 240. 2000; N.P.Singh et al., Fl. Bihar, Analysis 114. 2001; T.K.Sarma & A.K.Sarkar, Fl. Palamau Dist. 156. 2002; K.P.Singh in N.P.Singh et al. (Eds), Fl. Mizoram 1: 388. 2002; G.D.Pal, Fl. Lower Subansiri Dist. 1: 215. 2103, excl. syn. L. trifoliata; B.K.Shukla in K.P.Singh et al. (Eds), Fl. Uttar Pradesh 1. 351. 2016, excl. syn. L. trifoliata; S.S.Dash & P.Singh, Fl. Kurung Kumey Dist. 384. 2017, excl. syn. L. trifoliata; Pusalkar & S.K.Srivast., Fl. Uttarakhand 1: 989. 2018. Type: INDIA. Nagaland: Naga hills, 3 Nov 1836, Griffith Herb. Propr. N. 1297 (neotype, K K004252754, here designated).
Leea bracteata C.B.Clarke, Trimen’s J. Bot. 19: 164. 1881; Kanjilal & Das, Fl. Assam 1(2): 308. 1936; Deb, Fl. Tripura 1: 419. 1981; A.K.Mukh., Pachmarhi & Bori Reserves 68. 1984. Type: INDIA. Sikkim, s.d., Hook.f. & Thomson 169 (lectotype, designated by Ridsdale 1974, pg. 88: K [K001615004]).

##### Description.

Erect subshrub or shrub, 1–2 m high; roots non-tuberous. Stems erect or straggling, stout, jointed, with branches, young stem hispid or pubescent; internodes furrowed or round, green; nodes not swollen; pearl glands globose, more on inflorescences, white. Leaves alternate, pinnate to bipinnate, imparipinnate; petioles 7–21 mm long, base bulged, sheathing the stem, glabrous, green. Petiolar stipules narrowly winged, 4–8 cm long, extending to half of petioles; stipular scars triangular; rachises 5–31 cm long, channeled on upper surface, hispid, green. Leaflets opposite, ovate widely ovate or obovate, distal leaflets: lamina 14–32 × 6.5–13.5 cm, lateral leaflets: lamina 7.5–30 × 4–13 cm, glabrous, young leaves green or brown, mature leaves green on upper surface and pale green on lower, base cuneate to rounded, margins serrate, apex long acuminate or caudate; petiolules: central 3–8 cm long, green, lateral 0.4–2.5 cm long; secondary veins 7–16 pairs, 3-nerved at base, vein raised on abaxial side. Inflorescences terminal, a leaf-opposed compound cyme, 8–22 cm long, many flowered, condensed, hispid; peduncles 1–4 cm long, furrowed, pubescent, green, hispid. Flowers 4–6.2 mm, actinomorphic, greenish with brown tinges. Pedicels slender, 1.5–2 mm long, hispid. Bracts 3.6–8.3 × 1.9–5 mm, broadly ovate, conspicuous, persistent, green, hispid. Calyx campanulate, 3.5–5.6 × 3–3.3 mm, 5 lobed, lobes 0.9–1.1 × 0.75–1.9 mm, ovate or widely ovate, apex acute, green, hispid; corolla lobes 5, ovate-oblong, 2.7–3.5 × 1.9–2.4 mm, apex acute, hooded, margin entire, glabrous, green; corolla tube with floral disc 3.1–3.3 × 3–3.2 mm. Floral disc 1.6–2.4 mm long, orange-red; upper free part 1.1–1.8 mm long, notched; sinuses shallow, 0.25–0.4 mm long; lower free part 0.8–1 mm long. Stamens 5, syngenecious; filaments 1.25–1.3 mm long, glabrous, white; anthers 1.2–1.3 mm long, dehiscing longitudinally, purple on backside, creamish on margin. Ovary globose, 0.95–1.1 mm long, superior, 4–6-loculed, one ovule in each locule, placentation axile; style slender, 1.1–2.2 mm long, glabrous, creamy; stigma round. Fruits a berry, yellow when young, turning purple black at maturity, 6-seeded. Seeds wedge-shaped, 4.72–4.91 × 3.72–3.85 mm, light brown when mature.

**Figure 4.**
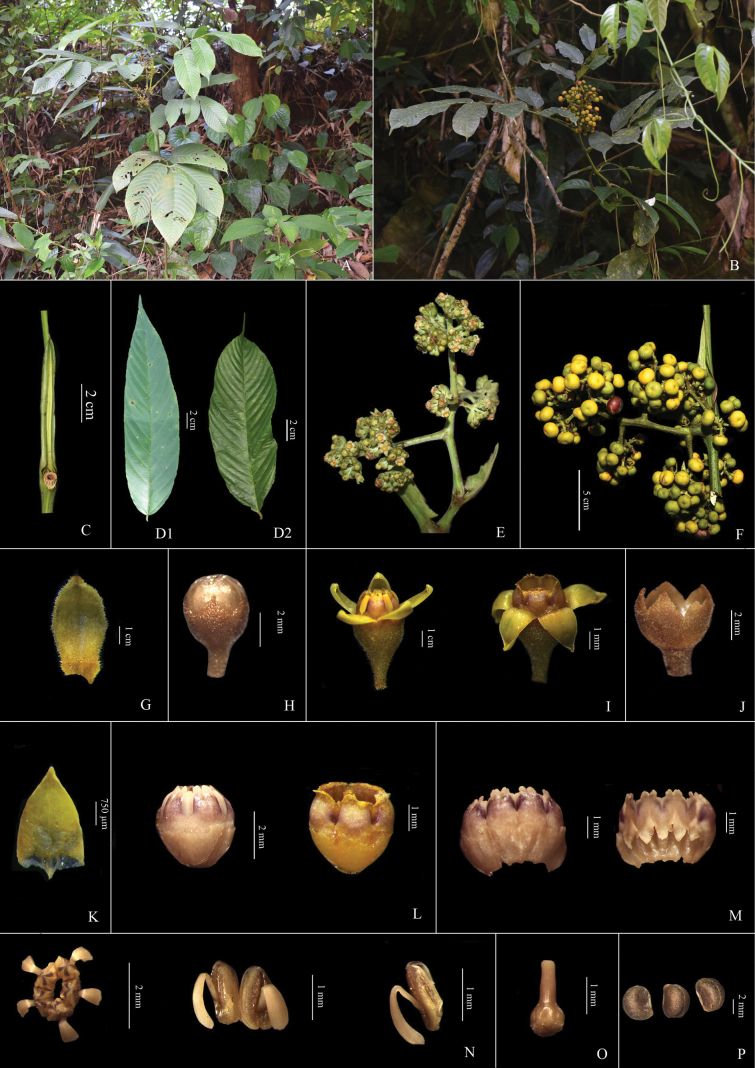
*Leea compactiflora* Kurz: **A.** Habit; **B.** Fruiting twig; **C.** Petiolar stipule; **D1, D2.** Leaflets; **E.** Inflorescence; **F.** Infructescence; **G.** Bract; **H.** Flower bud; **I.** Flower; **J.** Calyx; **K.** Corolla lobe; **L.** Corolla tube with floral disc; **M.** Corolla tube with floral disc split open; **N.** Stamens; **O.** Pistil; **P.** Seeds.

##### Flowering and fruiting.

May–November.

##### Habitat.

This species is usually found growing near roadsides and forest margins. The common associates are *Elatostemma* sp., *Boehmeria glomerulifera* Miq. (both Urticaceae), *Chromolaena odorata* (L.) R.M.King & H.Rob. (Asteraceae) and some ferns and grasses.

**Figure 5.**
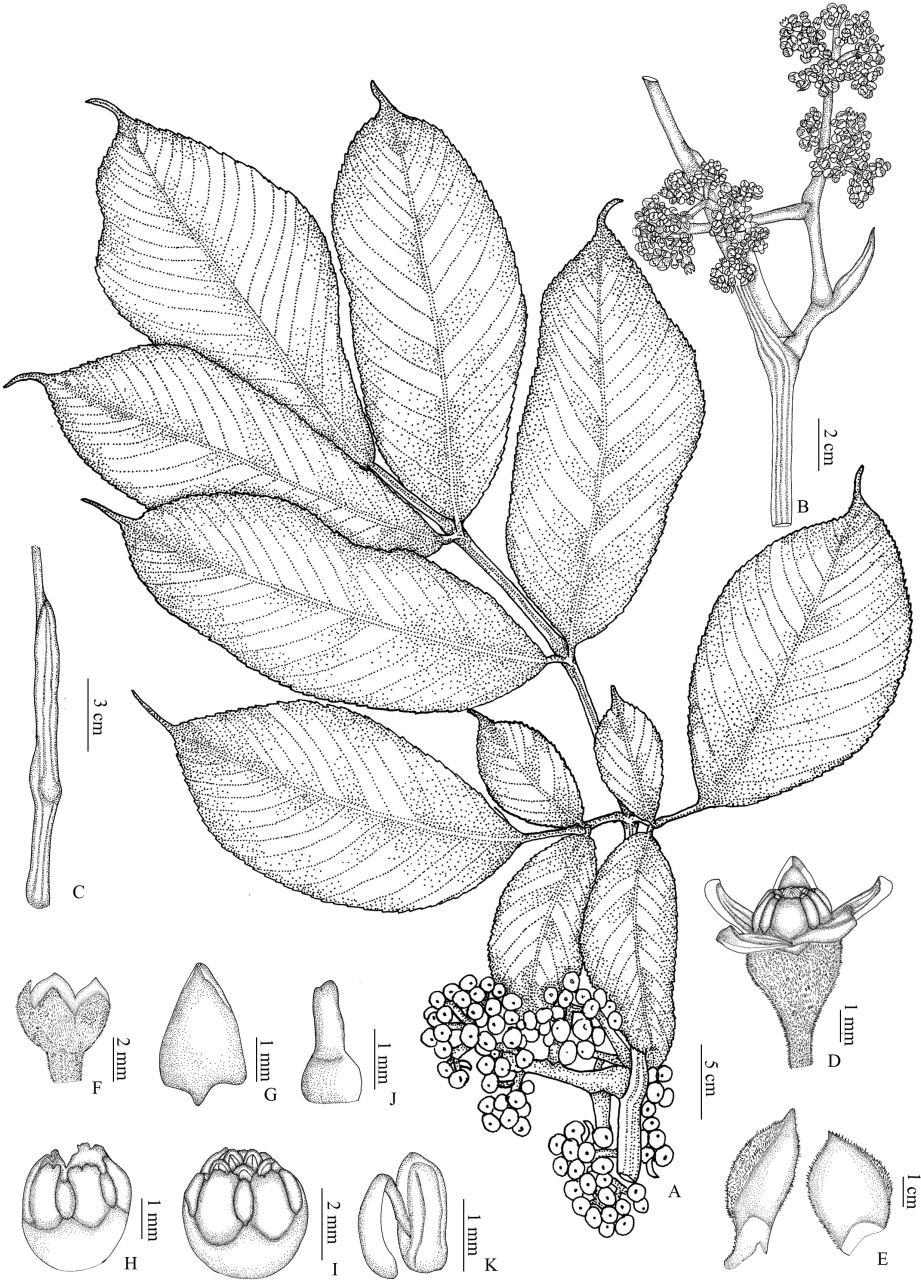
*Leea compactiflora* Kurz: **A.** Fruiting twig; **B.** Inflorescence; **C.** Petiolar stipule; **D.** Flower; **E.** Bract; **F.** Calyx; **G.** Corolla lobe; **H, I.** Corolla tube with floral disc; **J.** Pistil; **K.** Stamens (Drawn by Amrutha A. from Amrutha 159829).

##### Distribution.

**Figure 6.**
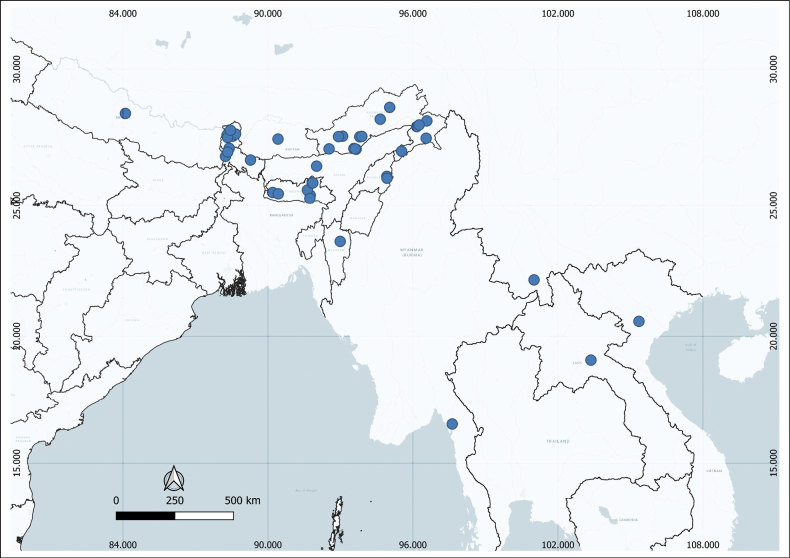
Distribution of *Leea compactiflora* Kurz (drawn using QGIS version 3.24.3).

India (Arunachal Pradesh, Assam, Bihar, Manipur, Meghalaya, Mizoram, Sikkim, Uttarakhand, Uttar Pradesh and West Bengal), Bhutan, China, Laos, Myanmar, Nepal and Vietnam. Fig. 6.

##### Specimens examined.

**China. Yunnan province**: southern Yunnan between Keng Hung [Jinghong] and Muang Hing [Meng Hai], 02-25-1922-01-03-1922, J.F. Rock 2642 (US 1213169!). **India.** • **Arunachal Pradesh**: Anjaw district, Hayuliang-Borpu, 25 Nov 1957, R.S. Rao 10819 (CAL); • Changlang district, Namdapha National Park, 05 Aug 2018, Amrutha 159829 (CALI); • East Siang district, Rengging, 24 Feb 1912, I.H. Burkill 36711 (CAL); • Rotung, 31 Dec 1911, I.H. Burkill 37599 (CAL); • Lohit district, Way to Tidding from Tezu, 10 Aug 2018, Amrutha 159836 (CALI); • Lower Subansiri district, Innerline beyond Kimin River up to 1 mile along the road to Ziro, 23 Nov 1957, G. Panigrahi 11469 (CAL); • Way to Raga, 16 Aug 2018, Amrutha 159850 (CALI); • Papum Pare district, 5 km from Chimpu, 03 Aug 1994, G.D. Pal 6709 (ARUN); • Itanagar-Hydel, 25 Sept 1978, G.D. Pal 76051 (ARUN); • Senki view, Itanagar, 27°06'13.7"N, 93°36'38.2"E, 300 m, 30 May 2022, Amrutha 164710 (CALI); • Tirap district, Lai Longsong, 19 June 1961, D.B. Deb 25781 (CAL); • Rusa-Bimalpur, 07 Sept 1958, G. Panigrahi 17025 (CAL); • Upper Siang district, Way to Hawa Camp, Yingkiong, 02 Aug 2018, Amrutha 159865 (CALI); • West Kameng district, Sessa Village, 01 June 2022, Amrutha 164722 (CALI); • West Siang district, Kane WLS, 13 Sept 2008, S.S. Dash 32228 (ARUN); • **Assam**: Darrang district, Darrang, 22 Apr 1914, Upendranath Kanjilal s.n. (ASSAM); • **Meghalaya**: East Khasi Hill district, Khasi hills, 30 Oct 1872, Clarke, C.B. 17726F (K004252751); • Ibid., 0-4000 ft, s.d., J.D. Hooker & J. J. Thomson s.n. (K K004252741, K004252745, K004252749); • Ri-Bhoi district, Balaiba Tilla, Nongpoh, 12 May 1965, J. Joseph 42328 (ASSAM); Nongpoh, 2000 ft, 02-15, May 1945, Thakur Rup Chand 1504 (MICH 1506767; L 0532034); • Ibid., 2000 ft, 16 Jun 1949, Thakur Rup Chand 22982 (MICH 1006084 A & B; L 0532033); Ibid., 13 May 1914, Upendranath Kanjilal 3977 (ASSAM); • Sohra, 4000ft, 24 May 1952, Walter N. Koelz 30039 (MICH 1506768); • West Garo Hills district, 1 Km after entry gate, foothills of Tura Peak, 25°29'41.7"N, 90°12'02.4"E, 03 June 2022, Amrutha 164735 (CALI); • Rongsingiri, Nokrek Biosphere Reserve, 17 Oct 2007, V.N. Singh & Bikarma Singh 115870 (ASSAM); • **Mizoram**: Aizawl district, Seling, Aizawl Seling-Champhai Road, 800 m, 07 June 2022, Amrutha 164748 (CALI); • **Nagaland**: Naga hills, 17 Oct 1885, Clarke, C.B. 40845A (K K004252750); Jabocka, Naga Hills, Apr 1899, Dr. Prains Collector 907 (CAL); • **Sikkim**: s.d., Kurz s.n. (CAL); • Ibid., 11 Jan 1874, G. King 839 (CAL); • Ibid., 24 Aug 1875, King, G 2324 (L 0744659); • Ibid., Regio trop., 1-6000 feet, J.D. Hooker s.n. (K004252736); • Ibid., 1-5000 feet, J.D. Hooker s.n. (K004252729, K004252730, K004252731, K004252732, K004252735, K004252758; P02342891); • Sikkim Himalaya, 1881, G. King s.n. (US 01151425!); Gangtok district, Singtam, 18 May 1967, N.C. Mazumder & R.M. Dutta 269 (CAL); • **West Bengal**: Darjeeling district, Rishop, 5 June 1870, Clarke, C.B. 11824a, (K K004252737); Jalpaiguri district, Dayamara, Jaldapara National Park, 09 May 2014, K. Karthigeyan 61470 (CAL barcode CAL0000209976); • Rungbee, 8 Oct 1908, W.W. Smith 282 (CAL). **Laos.** Sekong province, shrub in forest, 28 Apr 2013, fl, H. Sun et al. 14995 (KUN).

##### Conservation status.

In India, this species is found in Northeast India, especially in Arunachal Pradesh. The Extent of Occurrence (EOO) is calculated to be 1417,687 Km^2,^ and the Area of Occupancy (AOO) is 164.00 Km^2^. They are mostly seen in forest margins and on the roadside. Because of its number of populations and localities, this species can be considered as Least Concern (LC) according to the IUCN Standards and Petitions Committee (2022).

##### Notes.

*Leea compactiflora* was first described by Kurz (1873) with a very short diagnosis, and he noted that the specimen was collected from Martaban, Myanmar. After an exhaustive search in various herbaria, we could not locate the sheet of Kurz from Martaban. Ridsdale (1974) also indicated that he did not trace the material. Therefore, a neotypification is required as per Art. ICN 9.8. Here, we select a specimen from the Naga hills, India, collected by Griffith, housed at the Kew herbarium (K004252754) as the neotype following the Madrid Code (Turland et al. 2025), as it matches the protologue and was cited by Clarke (1881) in his revision of Indian species of *Leea*.

## Supplementary Material

XML Treatment for Leea trifoliata

XML Treatment for Leea compactiflora

## ﻿Additional information

### Use of AI

No use of AI was reported.

### Author ORCIDs

Amrutha Athalappil https://orcid.org/0000-0002-3579-0060

Ayilliath Kuttiyeri Pradeep https://orcid.org/0000-0003-0994-5599

Jun Wen https://orcid.org/0000-0001-6353-522X

Nikhil Krishna https://orcid.org/0000-0002-2616-9854

### Data availability

All of the data that support the findings of this study are available in the main text.

## References

[B1] APG III [Angiosperm Phylogeny Group] (2009) An update of the Angiosperm Phylogeny Group classification for the orders and families of flowering plants: APG III. Botanical Journal of the Linnean Society 161: 105–121. 10.1111/j.1095-8339.2009.00996.x

[B2] APG IV [Angiosperm Phylogeny Group] (2016) An update of the Angiosperm Phylogeny Group classification for the orders and families of flowering plants: APG IV. Botanical Journal of the Linnean Society 181: 1–20. 10.1111/boj.12385

[B3] Beentje HJ (2016) The Kew plant glossary: an illustrated dictionary of plant terms. Royal Botanic Gardens, Kew. Kew Publishing, Richmond, UK.

[B4] Chen ZD Wen J (2007) Leeaceae. In: Wu CY Hong DY Raven PH (Eds) Flora of China.Vol. 12. Science Press, Beijing & Missouri Botanical Garden Press, St. Louis, 169–173.

[B5] Clarke CB (1881) A revision of the Indian species of *Leea*. Journal of Botany 19: 100 106, 135–142, 163–167.

[B6] Gagnepain F (1950) Leeacées. In: Gagnepain F (Ed.) Flore générale de l’Indo-Chine.Suppl. Vol. 7. Masson, Paris, 844–855.

[B7] Gamble JS (1918) Flora of the Presidency of Madras. Vol. 1. Adlard & Sons Ltd., London. 10.5962/bhl.title.21628

[B8] Harris JG Harris MW (1995) Plant identification terminology, an illustrated glossary. (2^nd^ edn.). Spring Lake Publishing, Payson, Utah.

[B9] IUCN Standards and Petitions Committee (2022) Guidelines for Using the IUCN Red List Categories and Criteria. Version 15.1. Prepared by the Standards and Petitions Committee. https://www.iucnredlist.org/documents/RedListGuidelines.pdf

[B10] Kanjilal UN Das A (1936) Flora of Assam. Vol. 1. Part ii. Government of Assam, 303–308.

[B11] Kurz S (1873) New Burmese plants part II. Journal of the Asiatic Society of Bengal 42(2): 59–110.

[B12] Latiff A (2001) Diversity of the Vitaceae in the Malay Archipelago. Malayan Nature Journal 55(1 & 2): 29–42.

[B13] Lawson MA (1875) *Leea*. In: Hooker JD (Ed.) Flora of British India.Vol. 1. L. Reeve & Co., London, 664–668.

[B14] Lok AFSL Ang WF Ng BYQ Suen SM Yeo CK Hugh TWT (2011) *Leea* L. (Vitaceae) in Singapore. Nature in Singapore 4: 55–71.

[B15] Molina JE Wen J Struwe L (2013) Systematics and biogeography of the non viny grape relative *Leea* (Vitaceae). Botanical Journal of the Linnean Society 171(2): 354–376. 8339.2012.01320.x

[B16] Naithani BD (2000) Leeaceae. In: Singh NP Vohra JN Hajra PK Singh DK (Eds) Flora of India.Vol. 5. Botanical Survey of India, Kolkata, 325–342.

[B17] Planchon JE (1887) Monographie des Ampélidées vraies. In: de Candolle ACP de Candolle ALPP (Eds) Monographiae Phanerogamarum.Vol. 5. Masson, Paris, 305–610.

[B18] POWO (2025) Plants of the World Online. – Facilitated by the Royal Botanic Gardens, Kew. [retrieved 17 September 2025]

[B19] QGIS (Quantum GIS Development Team) (2022) Quantum GIS geographic information system. http://download.qgis.org

[B20] Ren H Pan KY Chen ZD Wang RQ (2003) Structural characters of leaf epidermis and their systematic significance in Vitaceae. Zhiwu Fenlei Xuebao 41: 531–544.

[B21] Ridsdale CE (1974) A revision of the family Leeaceae. Blumea 22(1): 57–100.

[B22] Ridsdale CE (1976) Leeaceae. Flora Malesiana. Series. 1(7): 755–782.

[B23] Simpson MG (2019) Plant systematics. 3^rd^ edn. Academic Press. 10.1016/B978-0-12-812628-8.50001-8

[B24] Stearn WT (1983) Botanical Latin-History, Grammar, Synyax, Terminology and Vocabulary. David & Charles, London.

[B25] Suessenguth K (1953) Leeaceae. In: Engler A Prantl K (Eds) Die natürlichen Pflanzenfamilien, 2nd edn., 20d. Duncker & Humbolt, Berlin, 372–390.

[B26] Thiers B (2024) continuously updated: Index Herbariorum: A global dictionary of public Herbaria and associated staff. New York Botanical Garden’s virtual herbarium. http:sweetgum.nybg.org/science/ih/ [accessed on 19.09.2025]

[B27] Turland NJ Wiersema JH Barrie FR Gandhi KN Gravendyck J Greuter W Hawksworth DL Herendeen PS Klopper RR Knapp S Kusber WH Li DZ May TW Monro AM Prado J Price MJ Smith GF Señoret JCZ [Eds] (2025) International Code of Nomenclature for algae, fungi, and plants (Madrid Code) adopted by the Twentieth International Botanical Congress, Madrid, Spain, in July 2024. Regnum Vegetabile 162. University of Chicago Press, Chicago, USA.

[B28] Wen J (2007a) Leeaceae. In: Kubitzki K (Ed.) The Families and Genera of Vascular Plants.Vol. 9. Springer-Verlag, Berlin, 221–225.

[B29] Wen J (2007b) Vitaceae. In: Kubitzki K (Ed.) The Families and Genera of Vascular Plants.Vol. 9. Springer-Verlag, Berlin, 467–479.

